# Evaluation of Image Reconstruction Algorithms for Confocal Microwave Imaging: Application to Patient Data

**DOI:** 10.3390/s18061678

**Published:** 2018-05-23

**Authors:** Muhammad Adnan Elahi, Declan O’Loughlin, Benjamin R. Lavoie, Martin Glavin, Edward Jones, Elise C. Fear, Martin O’Halloran

**Affiliations:** 1 Electrical and Electronic Engineering, National University of Ireland Galway, H91 TK33 Galway, Ireland; d.oloughlin@outlook.com (D.O’L.); martin.glavin@nuigalway.ie (M.G.); edward.jones@nuigalway.ie (E.J.); martin.ohalloran@nuigalway.ie (M.O’H.); 2 Department of Electrical and Computer Engineering, University of Calgary, Calgary, AB T2N 1N4, Canada; brlavoie@ucalgary.ca (B.R.L.); fear@ucalgary.ca (E.C.F.)

**Keywords:** microwave imaging, ultra wideband radar, breast cancer, artifact removal, patient study

## Abstract

Confocal Microwave Imaging (CMI) for the early detection of breast cancer has been under development for over two decades and is currently going through early-phase clinical evaluation. The image reconstruction algorithm is a key signal processing component of any CMI-based breast imaging system and impacts the efficacy of CMI in detecting breast cancer. Several image reconstruction algorithms for CMI have been developed since its inception. These image reconstruction algorithms have been previously evaluated and compared, using both numerical and physical breast models, and healthy volunteer data. However, no study has been performed to evaluate the performance of image reconstruction algorithms using clinical patient data. In this study, a variety of imaging algorithms, including both data-independent and data-adaptive algorithms, were evaluated using data obtained from a small-scale patient study conducted at the University of Calgary. Six imaging algorithms were applied to reconstruct 3D images of five clinical patients. Reconstructed images for each algorithm and each patient were compared to the available clinical reports, in terms of abnormality detection and localisation. The imaging quality of each algorithm was evaluated using appropriate quality metrics. The results of the conventional Delay-and-Sum algorithm and the Delay-Multiply-and-Sum (DMAS) algorithm were found to be consistent with the clinical information, with DMAS producing better quality images compared to all other algorithms.

## 1. Introduction

Confocal Microwave Imaging (CMI) is an emerging imaging modality for the detection of breast cancer. One important signal processing challenge in reconstructing high quality breast images using CMI is the image reconstruction algorithm itself. An effective image reconstruction algorithm provides an accurate localisation of tumours, while suppressing clutter due to healthy breast tissues and any residual artifacts from preprocessing.

Several image reconstruction algorithms for CMI have been developed over the last two decades [1,2,3,4,5,6,7,8,9,10,11,12,13]. These algorithms have been categorised as Data Independent (DI) beamforming and Data Adaptive (DA) beamforming algorithms in the literature [14]. Both DI and DA beamforming algorithms are based on the principle of coherent addition of backscattered radar signals, which are collected after illuminating the breast with Ultra-Wideband Radar (UWB) pulses. In Data-Independent (DI) beamforming algorithms, coherent addition is performed based on an assumed propagation model. However, Data Adaptive (DA) algorithms estimate the propagation model from the received signals and apply compensation factors based on this estimated channel model. Several studies have been performed to evaluate the performance of both DI and DA beamforming algorithms using a variety of numerical and physical breast models [7,12,15,16,17,18,19,20,21,22]. However, these studies have often evaluated the performance of both DI and DA algorithms using a limited set of beamforming algorithms [7], simplified numerical phantoms [12,19,20], and an idealised artifact removal algorithm, while ignoring the impact of realistic artifact removal [15,16,18]. One recent study applied multiple imaging algorithms to clinical data from healthy volunteers [21]. The volunteers had no breast cancer so tumour responses were artificially introduced in the acquired data. Differential signals, derived from these signals containing artificially induced tumour responses, were used for imaging. The results from this clinical investigation are, thus, inherently based on several assumptions, such as the availability of accurate tumour signature templates and baseline measurements. Therefore, these results may not be generalisable to the diagnostic application where baseline measurements are not available, and where an accurate estimation of the tumour signature template in an unknown breast density is not possible. In conclusion, the current literature lacks a comprehensive evaluation of a variety of imaging algorithms in realistic clinical scenarios [23].

In this study, the performance of a variety of DI and DA algorithms is evaluated using clinical patient data. The following algorithms are examined in this study:

- Delay-And-Sum (DAS)
- Improved Delay-And-Sum (IDAS)
- Delay-Multiply-And-Sum (DMAS)
- Coherence Factor Based DAS (CF-DAS)
- Channel Ranked DAS (CR-DAS)
- Robust Capon Beamformer (RCB)

The novelty of this study is two-fold: firstly, a comprehensive set of both DI and DA beamforming algorithms was examined; and, secondly, these algorithms were evaluated together for the first time on both clinical patient data and with a non-idealised artifact removal algorithm. The patient data used in this study were obtained from a first patient study conducted at the University of Calgary [24]. The patients were scanned using the first generation Tissue Sensing Adaptive Radar (TSAR) prototype [25]. In the original publication [24], breast images were reconstructed using the traditional DAS algorithm. The images reconstructed with the DAS algorithm were consistent with clinical information in most cases. However, not all lesions were detected. This current study aimed to evaluate more advanced imaging algorithms on the same patient data. Improved clutter suppression capabilities of the more advanced Confocal Microwave Imaging (CMI) algorithms are expected to improve upon the DAS imaging results for the University of Calgary patient study reported in [24].

The remainder of this paper is organised as follows: Section 2 provides an overview of the imaging algorithms that are considered in this study. Section 3 describes the patient details, patient scanning and preprocessing of the scanned data. Section 4 and Section 5 present results and discussions, respectively. Finally, conclusions are presented in Section 6.

## 2. Imaging Algorithms

The imaging algorithms evaluated in this study are described in this section.

### 2.1. Delay-And-Sum

The conventional DAS beamformer equation is given as:

(1) $$I\left(\vec{r}\right)={\left[\sum_{i=1}^{M}b_{i}\left(\tau_{i}\left(\vec{r}\right)\right)\right]}^{2}$$

where *M* is the total number of channels, $b_{i}$ is the backscattered signal recorded at Channel *i*, and $\tau_{i}\left(\vec{r}\right)$ is the time required to travel the round trip distance between focal point $\vec{r}=\left(x,y,z\right)$ and antenna *i*.

In the DAS beamformer, firstly, the signals received at individual channels are time-aligned for each focal point $\vec{r}$. The time-alignment process involves calculation of the time-delay $\tau_{i}\left(\vec{r}\right)$ for each individual backscattered signal. The time-delay for each signal received at antenna *i* is calculated based on the assumed average propagation speed in the breast and the round trip distance between the focal point $\vec{r}$ and antenna *i*. Next, the time-aligned signals are simply summed together. Reflections from any tumour present are expected to add coherently. Conversely, reflections from healthy tissues and any artifacts from preprocessing are expected to add incoherently. The energy of the summed signal (often computed over a time-window) is assigned to the intensity of the focal point under consideration and this process is repeated for all focal points within the 3D breast volume to create a 3D breast image.

### 2.2. Delay-Multiply-And-Sum

The DMAS beamformer is similar to the conventional DAS [26] with an additional multiplication step [27]. However, DMAS is expected to provide better clutter reduction, compared to DAS, which is achieved by virtually increasing the sample size using signal-pair multiplication.

In the DMAS algorithm, the signals are multiplied in pairs after time-alignment for each focal point $\vec{r}$ of the breast. The pairing multiplication step virtually increases the number of signals from *M* to ${}^{M}C_{2}$ (i.e., combinations of size 2 from total *M* signals). All the ${}^{M}C_{2}$ signals are summed together in the next step, and the summed signal is then integrated over a time-window to compute the energy corresponding to each focal point.

### 2.3. Improved Delay-And-Sum

The IDAS beamformer modifies the conventional DAS by introducing an additional weighting factor for each focal point [28]. The weighting factor rewards the coherent addition of signals based on the quality of coherence, and this weighting factor is termed “Quality Factor (QF)”. The QF is introduced to Equation (1) to produce the IDAS beamformer equation:

(2) $$I\left(\vec{r}\right)=QF\left(\vec{r}\right)\cdot {\left[\sum_{i=1}^{M}b_{i}\left(\tau_{i}\left(\vec{r}\right)\right)\right]}^{2}$$

The QF for each focal point $\vec{r}$ is calculated from an energy collection curve obtained from coherent summation of radar signals. Perfect coherent summation of radar signals is expected to result in an energy collection curve that resembles a quadratic curve. Therefore, a second-order polynomial $\left(y=ax^{2}+bx+c\right)$ is fitted to the normalised energy collection curve using least-square fitting. The energy collection curve is normalised by multiplication of the curve by $1/(1+\sigma_{e})$, where $\sigma_{e}$ is the standard deviation of the energy curve. Next, the coefficients of the second-order polynomial are estimated. The coefficient *a* is chosen as the QF of focal point $\vec{r}$, and is multiplied with the final energy computed for the focal point $\vec{r}$.

### 2.4. Coherence Fator Based Delay-And-Sum

In the CF-DAS beamformer, a different coherence based weighting factor is introduced in the conventional DAS algorithm [29]. The Coherence Factor (CF) was adapted from ultrasound imaging [30] and is used to measure and enhance the coherence quality of radar signals. The CF is calculated for each focal point $\vec{r}$ as:

(3) $$CF\left(\vec{r}\right)=\frac{{\left|\sum_{i=1}^{M}b_{i}\left(\tau_{i}\left(\vec{r}\right)\right)\right|}^{2}}{\sum_{i=1}^{M}{\left|b_{i}\left(\tau_{i}\left(\vec{r}\right)\right)\right|}^{2}}$$

Then, the intensity at focal point $\vec{r}$ can be calculated as:

(4) $$I\left(\vec{r}\right)=CF\left(\vec{r}\right)\cdot {\left[\sum_{i=1}^{M}b_{i}\left(\tau_{i}\left(\vec{r}\right)\right)\right]}^{2}$$

### 2.5. Channel Ranked Delay-And-Sum

In the CR-DAS beamformer, each individual signal is scaled with a weighting factor [8]. The weighting factor is based on the length of the propagation path between the focal point and each antenna. Channels with shorter propagation paths are assumed to have a relatively clear view of the focal point, are less likely to encounter heterogeneity, and are less affected by the attenuation and phase effects. Conversely, channels with relatively long propagation paths are more likely to encounter significant heterogeneity and suffer more attenuation as the UWB signal propagates to and from the focal point. Therefore, channels with shorter propagation paths are given higher weighting in the imaging reconstruction process. The weighting factor for CR-DAS is calculated as follows:

(5) $$w\left(i\right)=\frac{M-rank_{\left(i\right)}}{M(M+1)/2}$$

where *M* is the number of signals, $rank_{\left(i\right)}$ is a number from 1 to *M*, assigned to each signal based on that signal’s round trip propagation distance (signal with the shortest propagation distance is assigned a rank of 1). The weighting factor is applied to each signal prior to coherent summation and formation of the energy image as given below:

(6) $$I\left(\vec{r}\right)={\left[\sum_{i=1}^{M}w\left(i\right)\cdot b_{i}\left(\tau_{i}\left(\vec{r}\right)\right)\right]}^{2}$$

### 2.6. Robust Capon Beamformer

The RCB algorithm is based on the Standard Capon Beamformer (SCB) [11,12]. The SCB estimates the signal energy by adaptively selecting a weight vector for the received signals. The weight vector is chosen to minimise the beamformer output power subject to the constraint that the signal of interest (SOI) does not suffer any distortion. However, in practice, SCB suffers performance degradation from imprecise knowledge of the array steering vector, often caused by waveform distortions, antenna location uncertainties and time-delay roundoffs. The presence of coherent interferences also contributes to the performance degradation of SCB. To mitigate the performance degradation due to steering vector errors, RCB introduces an uncertainty set for the array steering vector.

Consider the following preprocessed signal vector for a focal point $r_{0}$:

(7) $$y\left(t\right)={\left[b_{1}\left(t\right)\;b_{2}\left(t\right)\cdots b_{M}\left(t\right)\right]}^{T},t=0,\cdots ,N-1$$

Each snapshot y(t) can be modelled as:

(8) y(t)=a(t)·s(t)+e(t)

where s(t) is the backscattered signal, a denotes the steering vector, and $e\left(t\right)=[e_{1}\left(t\right)\;e_{2}\left(t\right)\cdots e_{M}\left(t\right)]$ includes the noise and interference due to undesired reflections. Assuming proper time alignment and signal compensation, the steering vector can be assumed to be $a={\left[1,\cdots ,1\right]}^{T}$. With the knowledge of the steering vector, s(t) can be estimated from y(t).

RCB assumes that the true steering vector $\hat{a}\left(t\right)$ lies in the vicinity of the assumed steering vector, a and the only knowledge available about $\hat{a}\left(t\right)$ is that ${∥\hat{a}\left(t\right)-a\left(t\right)∥}^{2}\leq \epsilon$, where ϵ is a user defined parameter to describe the uncertainty of $\hat{a}\left(t\right)$ [11,12]. The steering vector $\hat{a}\left(t\right)$ can be determined using a covariance fitting approach described in [12].

Given $\hat{a}\left(t\right)$, the RCB problem can be formulated as:

(9) $$\underset{w}{min}w^{T}\hat{R}\;w\;subject\;to\;w^{T}\hat{a}=1$$

where $\tilde{R}$ is the sample covariance matrix given as:

(10) $$\tilde{R}\equiv \frac{1}{M}y\left(t\right)y^{T}\left(t\right)$$

and the solution to Equation (9) is given as:

(11) $$\hat{w}=\frac{{\hat{R}}^{-1}\;\hat{a}}{{\hat{a}}^{T}{\hat{R}}^{-1}}$$

The beamformer output can be written as a vector

(12) $$\hat{s}\left(t\right)={\left[{\hat{w}}^{T}\left(t\right)y\left(t\right)\right]}^{T}$$

Finally, the backscattered energy from location $r_{0}$ can be calculated as:

(13) $$I\left(\vec{r_{0}}\right)=\sum_{t=1}^{N}{\hat{s}}^{2}\left(t\right)$$

## 3. Patient Scanning and Preprocessing

In this section, details of patients, the patient scanning process, and the preprocessing of scanned data is detailed.

### 3.1. Patient Information

The patient data used in this study came from the microwave imaging patient study conducted at the University of Calgary [24]. This study was approved by the University of Calgary Conjoint Health Research Ethics board (E-22121) and all patients provided informed consent. The patient recruitment and the patient scanning using the TSAR prototype are detailed in [24]. Most patients recruited into the study had a suspicious region in one breast, which was identified through a mammogram or a clinical examination. The breast with the suspicious region was scanned with the TSAR system. Prior to the TSAR scan, each patient was also scanned with a contrast enhanced Magnetic Resonance Imaging (MRI) to aid in interpretation of the TSAR images.

In this study, five patient cases are presented in detail. Three of these five patients had clearly identified benign or malignant lesions, while two patients had no disease. Table 1 summarises the important clinical information of each patient including patient age, breast density, and disease type.

### 3.2. Patient Scanning

**Figure 1:**
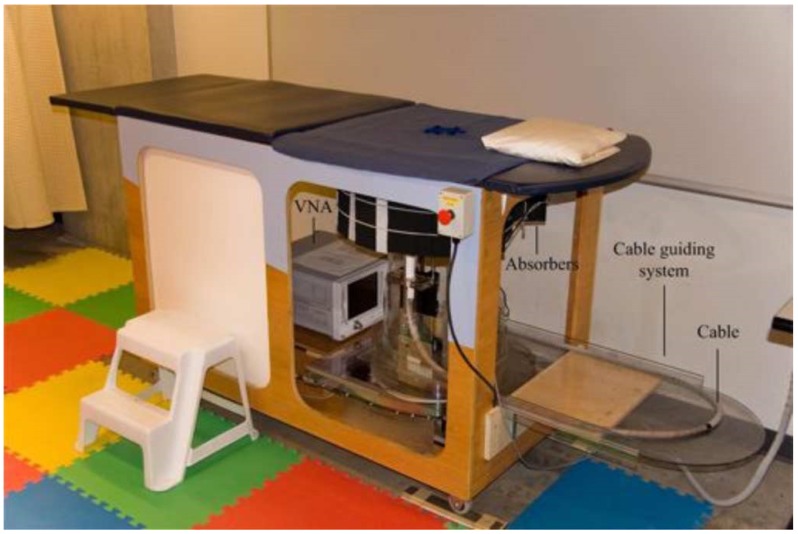
First generation Tissue Sensing Adaptive Radar Prototype (from [24]). For Figure 1, IEEE License received. License number 4274890619942.

The first generation TSAR prototype used for the patient scanning is described in [25]. The prototype (shown in Figure 1) features a monostatic data acquisition system with an UWB Balanced Antipodal Vivaldi Antenna with Director (BAVA-D) antenna [31] that is mounted on a positioning arm. To scan a breast, a patient lies on an examination table in the prone position and the measurements are collected by moving the arm vertically while the entire tank rotates. This results in a cylindrical scan configuration, where each measurement is taken by positioning the sensor at a fixed radius from the centre of the tank. The sensor positioning is controlled by actuating stepper motors using custom software. The prototype also has a laser system mounted on the positioning arm. The collected laser data are used to reconstruct the breast surface that can later be used to improve the reconstructed microwave images [32]. The reconstructed breast surface is also used to limit imaging domain to the surface of the breast under consideration. The system also has a digital camera to monitor the sensor positioning and the breast during the scanning process.

During the patient scan, patient lay in a prone position with one breast extended into the examination table of the prototype. Underneath the table, the BAVA-D antenna was vertically moved in a step of 5–10 mm between the nipple and chest wall of the breast. For each vertical position, multiple measurements were collected around the breast. The number of vertical positions (rows) for each patient was decided based on the vertical extent of the breast, which was estimated using the images acquired from the digital camera. For each patient, measurements were collected at up to 200 locations, as detailed in Table 1. The measurements were acquired using a Vector Network Analyser (VNA) at 1601 frequency points over the range of 50 MHz to 15 GHz with port power of −5 dBm. Intermediate Frequency (IF) bandwidth of 1 kHz and an average of three measurements was used to reduce the noise floor. For the calibration of the system, another set of measurements was collected without the patient present. The second set of measurements was collected at the same locations that were used to collect the patient measurements. These data were called a “calibration” scan.

### 3.3. Preprocessing

The frequency-domain data acquired from the VNA were preprocessed prior to imaging. Firstly, the calibration scan was subtracted from the patient scan. Next, the frequency-domain data were weighted with a differentiated Gaussian pulse, and converted to the time-domain using an inverse Chirp Z-transform [33]. The centre frequency of the pulse was 4 GHz and the full-width half-maximum frequency content was between 1.3 and 7.6 GHz. A phase-shift was also introduced in the calibrated data to compensate for the antenna aperture location. The resultant time-domain signals were then processed through the Neighbourhood-Based Skin Subtraction (NSS) algorithm for skin subtraction [34]. The NSS algorithm estimates the artifact at a particular antenna from a neighbourhood of antennas, and the estimated artifact is then subtracted from the target antenna.

## 4. Results

In the current study, the preprocessed patient data were used to reconstruct the breast images of patients using the DAS, IDAS, CF-DAS, DMAS, CR-DAS and RCB imaging algorithms. An average breast permittivity of $\epsilon_{r}=9$ was used to estimate the propagation speed and the corresponding distance travelled in the interior of the breast during image reconstruction. This value of permittivity may not be an accurate estimate for all patients and particularly for heterogeneously dense breasts, but it was chosen to compare results with the previous study that used permittivity of $\epsilon_{r}=9$ [24]. The reconstruction results obtained from each individual patient using different imaging algorithms are described in this section.

Microwave images obtained from each imaging algorithm are shown for each patient in Figures 3–9. Table 2, Table 3, Table 4, Table 5 and Table 6 summarise the analysis and interpretation of the microwave images of each patient. Information contained in each column of tables is described below:

- The first column in each table lists the imaging algorithm used to image the patient.
- The second column lists clinically identified (CI) regions of interest (ROI) that were detected through either clinical imaging or clinical examination and are expected to be detected in the microwave images. Any additional high intensity (HI) regions identified in microwave images (MI) are also listed in this column.
- The third column indicates whether CI-ROIs were detected in microwave images (MI).
- The fourth column of the table lists the Signal-to-Mean Ratio (SMR) of each high intensity region detected in the microwave images. The SMR is a measure of the quality of the beamformed image that provides a measure of separation between the ROI and the background clutter. It is defined as the ratio of the average intensity of the ROI to the average intensity of the overall 3D image.
- The fifth column lists the Full-width Half Maximum (FWHM) of each high intensity region detected in the microwave images. The FWHM may be used to estimate the extent of the ROI in the image. The FWHM is defined as twice the distance from peak intensity in the ROI to the point where intensity of ROI drops by half. The FWHM is computed by growing a region around the centroid of the ROI until the ROI intensity drops by half. Twice the average Euclidean distance from the centroid of the ROI to the end of the region is estimated to be the FWHM.
- The last column ranks the performance of each algorithm in terms of the detection of the CI-ROI and the quality of the image.

The results of each individual patient are discussed in the following sections.

### 4.1. Patient 1

**Figure 2:**
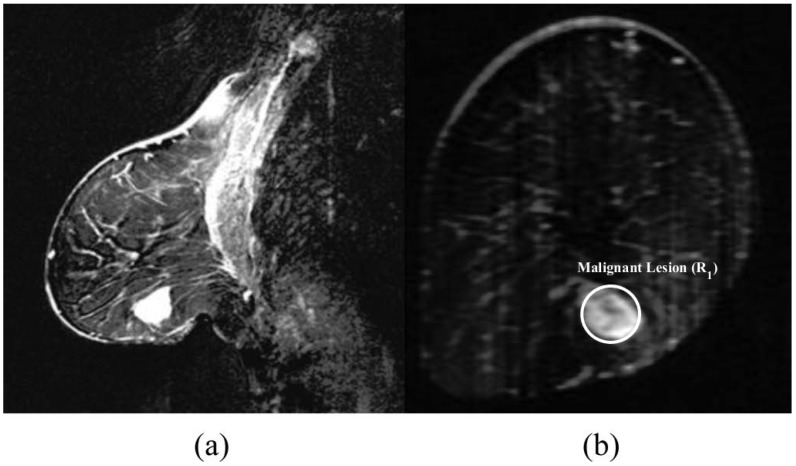
MRI scan of Patient 1 (from [24]): (**a**) saggital view; and (**b**) coronal view. For Figure 2, IEEE License received. License number 4274890619942.

**Figure 3:**
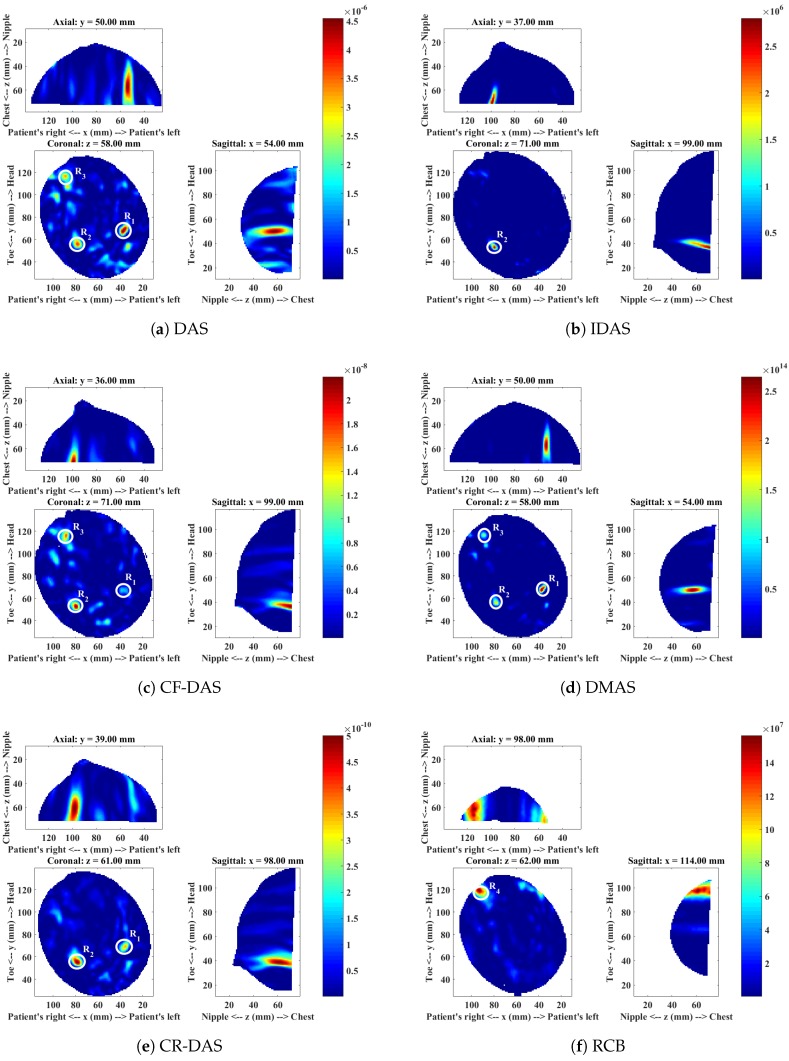
Microwave images of Patient 1 for a range of different imaging algorithms.

The mammogram indicated the presence of a 10 mm lesion at the 4 o’clock radian in the right breast of this patient. The MRI report showed the lesion at 5 o’clock, and a second possibly benign lesion at 7 o’clock. A Magenetic Resonance (MR) image of the patient is shown in Figure 2. The microwave images obtained from each imaging algorithm are shown in Figure 3 and the analysis of each image is presented in Table 2.

The image produced with DAS (Figure 3a) shows three responses $R_{1}$, $R_{2}$ and $R_{3}$. The dominant response $R_{1}$ at around the 5 o’clock position in the coronal slice corresponds to the location of the malignant lesion. The second response $R_{2}$ near to the 7 o’clock radian corresponds to the benign lesion, and the third response $R_{3}$ at approximately 11 o’clock corresponds to the fibroglandular concentration. 

The image obtained with the IDAS algorithm shows only one response $R_{2}$, which is dominant in the image. The dominant response corresponds to the location of the benign lesion. The other two responses $R_{1}$ and $R_{3}$ are low in intensity and are not clearly visible in the image. The CF-DAS algorithm also showed three responses with a dominant response $R_{2}$ at the location of the benign lesion. The response $R_{1}$, which corresponds to the malignant lesion is also present in the image. However, the intensity is much lower than both $R_{2}$ and $R_{3}$. The DMAS image shows all three responses. The location of the all three responses in the DMAS image are consistent with the DAS image, and are also consistent with the clinical information. The CR-DAS shows two responses, $R_{1}$ and $R_{2}$, where $R_{2}$ is the dominant response, which corresponds to the benign lesion. The dominant response $R_{4}$ in the RCB image is closer to the skin and does not correspond to any of the responses identified in images produced with other algorithms.

In terms of image quality, the IDAS, CF-DAS and CR-DAS have improved the SMR of $R_{2}$ when compared to DAS, where $R_{2}$ corresponds to the benign lesion. The DMAS algorithm has improved the SMR of all lesions. All algorithms have less average clutter compared to the DAS algorithm. However, DMAS has not only much less average clutter than all other algorithms but the dominant response also corresponds to the location of the malignant lesion, with a much higher SMR value than the SMR achieved by the DAS algorithm. DMAS also has consistent locations of all three responses with the DAS image and clinical information. The presence of a dominant scatterer near the skin in the RCB image indicates that the RCB has most likely enhanced early-time clutter.

Table 2 summarises the detection and the image quality metrics computed for each algorithm. The algorithms are ranked based on the detection of a malignant tumour and the SMR of the malignant tumour. DMAS has detected the malignant tumour with the highest SMR compared to all other algorithms. Therefore, it is ranked 1. The IDAS and RCB have failed to detect the malignant tumour. Hence, these algorithms have not been considered for ranking.

### 4.2. Patient 2

**Figure 4:**
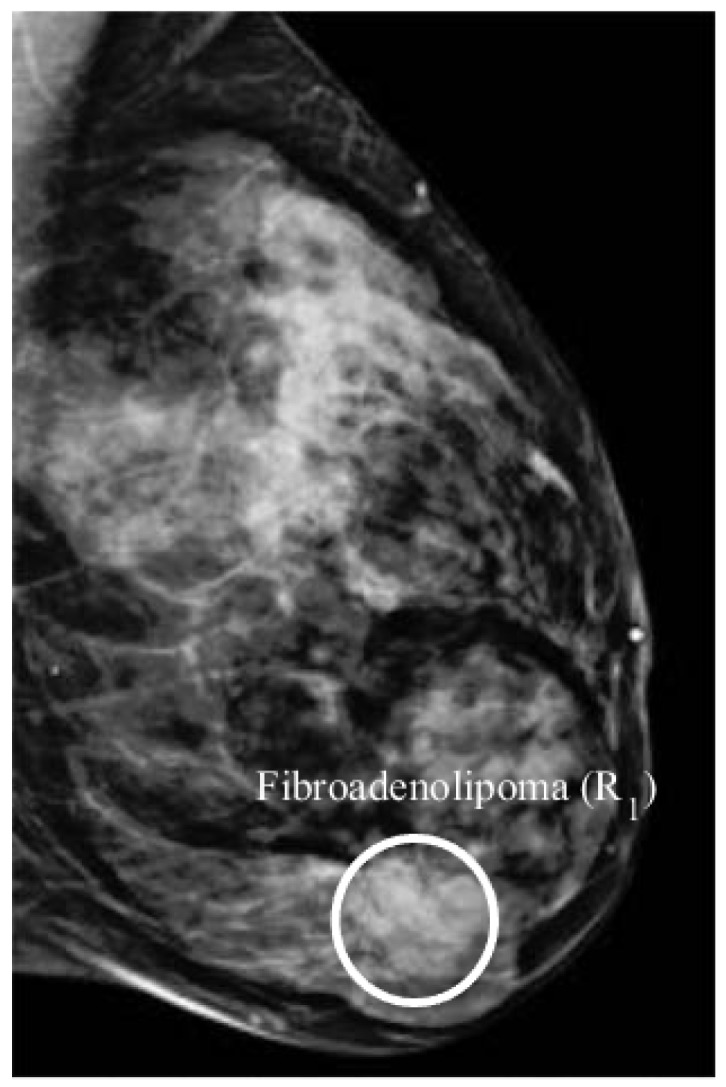
Mammogram of Patient 2 (from [24]). For Figure 4, IEEE License received. License number 4274890619942.

The mammogram of this patient reported a fibroadenolipoma of several centimetres in diameter, in the inner lower quadrant of the left breast (Figure 4). Fibroadenolipoma is a benign lesion formed with fibrous, glandular and fatty tissues encapsulated in a thin layer of connective tissue.

**Figure 5:**
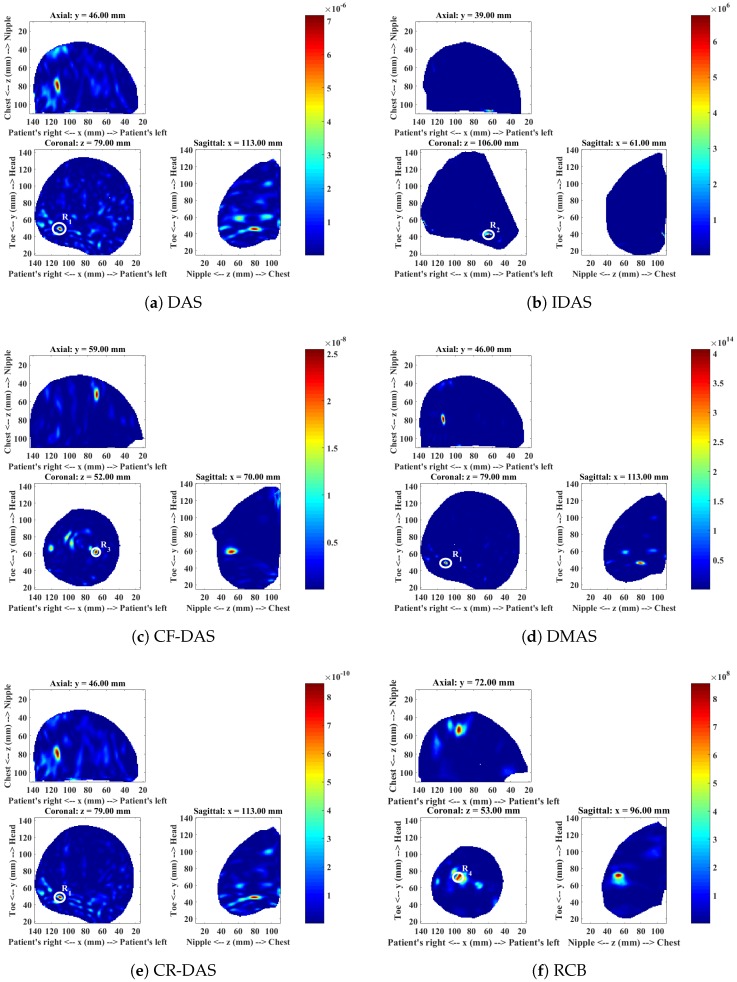
Microwave images of Patient 2 for a range of different imaging algorithms.

The microwave images of this patient are shown in Figure 5, and the analysis of the images is summarised in Table 3. The DAS image (Figure 5a) shows several responses in coronal slice. However, the dominant response $R_{1}$ is observed in the lower inner quadrant of the breast and is consistent with the clinical information. The dominant response in the IDAS image is closer to the chest wall and does not correspond to any known lesion in the breast. Similarly, the dominant response in the CF-DAS image is located in the lower outer quadrant and does not correspond to the clinically identified lesion. Similar to the DAS image, the DMAS image shows the dominant response in the lower inner quadrant of the breast. The CR-DAS has also produced an image that is similar to the DAS image, in terms of the location of the dominant response $R_{1}$. The dominant response in the RCB image is observed below the nipple and does not correspond to the known location of the fibroadenolipoma.

The images produced with the DAS, DMAS and CR-DAS are similar in terms of location of the dominant response and are also consistent with the clinical information. However, both the CR-DAS and the DMAS provide an improved SMR of the dominant response compared to the DAS algorithm, with the best SMR achieved with the DMAS algorithm. The IDAS, CF-DAS and the RCB algorithms suppress the average clutter in the images. However, the location of the dominant response is not consistent with the known location of the fibroadenolipoma.

Table 3 summarises the detection and the image quality metrics computed for each algorithm. The algorithms are ranked based on the detection of the benign lesion (the only CI-ROI) and the SMR of the benign lesion. The DAS, DMAS and CR-DAS algorithms have detected the benign lesion, with DMAS providing highest SMR. Therefore, it is ranked first. The IDAS, CF-DAS and RCB algorithms have not detected the lesion and therefore have not been considered in the ranking.

### 4.3. Patient 3

**Figure 6:**
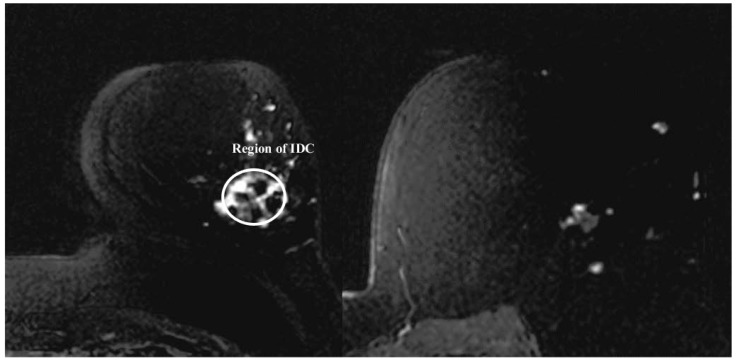
MRI scan of Patient 3 (from [24]). For Figure 6, IEEE License received. License number 4274890619942.

The MRI report of this patient showed enhancements from the 2 o’clock to 6 o’clock radians, and a focal mass located near the nipple (Figure 6). The mammogram showed extensive microcalcifications around the 3 o’clock position. The patient was later diagnosed with the Invasive Ductal Carcinoma (IDC) of size 14 cm × 2 cm × 2 cm in the upper outer quadrant of the breast.

**Figure 7:**
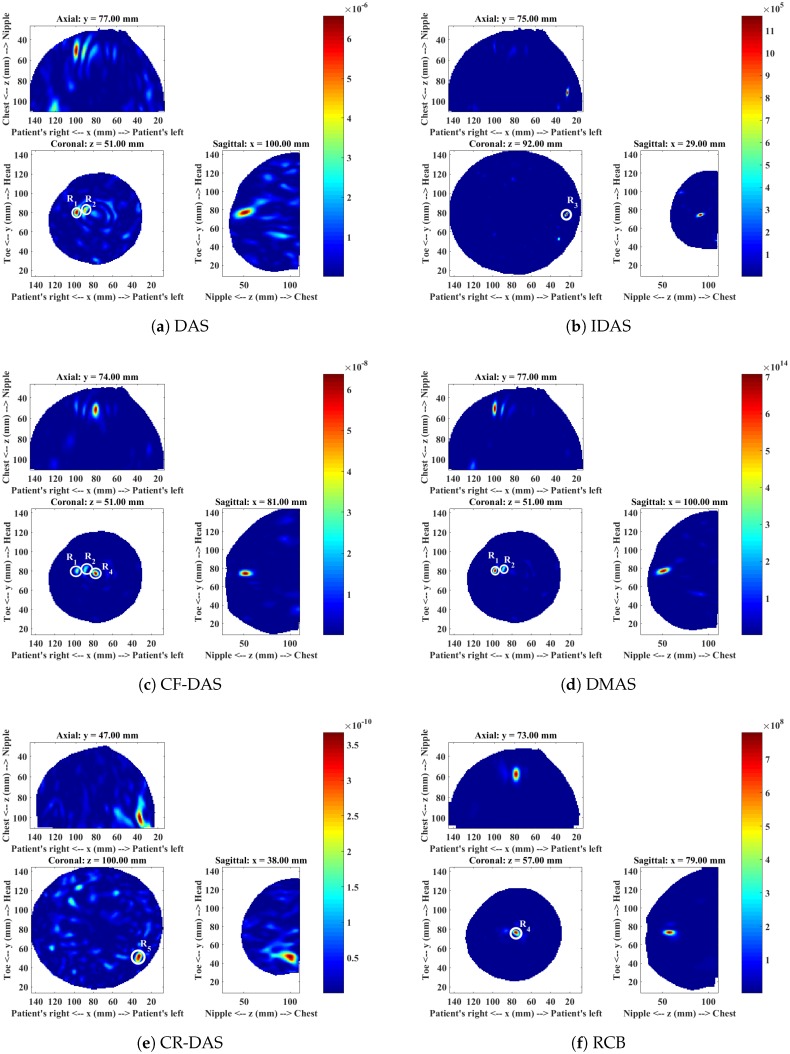
Microwave images of Patient 3 for a range of different algorithms.

The microwave images of this patient are shown in Figure 7. The analysis of each image is presented in Table 4. The DAS image shows two responses $R_{1}$ and $R_{2}$ near the nipple. These responses may correspond to a focal mass reported in the MRI report. The IDAS image does not show the responses $R_{1}$ and $R_{2}$ previously observed in the DAS image. The dominant response, $R_{3}$, in the IDAS image can be observed at approximately 3 o’clock (in the coronal view) and may correspond to the IDC. The CF-DAS image does show two responses $R_{1}$ and $R_{2}$ near the nipple. However, the dominant response $R_{4}$ is displaced compared to the DAS image. The DMAS image also shows two responses $R_{1}$ and $R_{2}$, as previously observed in the DAS image. The CR-DAS shows a dominant response $R_{5}$ at approximately 4 o’clock in the lower outer quadrant of the breast and does not correspond to any clinically identified lesion. The RCB image is similar to the CF-DAS image in terms of the dominant response location, but it only shows a single dominant response, $R_{4}$. The image produced with the IDAS has a dominant response at 3 o’clock, where it can possibly correspond to the IDC. The images obtained with the DAS, CF-DAS, DMAS and the RCB algorithms show responses near the nipple. As described earlier, these responses may correspond to the focal mass reported in the MRI. The images produced by DAS and DMAS are similar in terms of dominant response location, but DMAS has higher SMR of the dominant response and demonstrates better clutter suppression. Except IDAS, none of these algorithms have detected the IDC regions, as none of the dominant responses correspond to the IDC location.

Table 4 summarises the detection and the image quality metrics computed for each algorithm. The IDAS algorithm may have detected the IDC and also has shown the highest SMR. Therefore, IDAS has been ranked first. The other algorithms (DAS, CF-DAS, DMAS, CR-DAS and RCB) have not detected the IDC but they have shown the presence of the focal mass, which was reported in MRI. Therefore, the ranking of other algorithms has been based on the detection of the focal mass and the SMR of the focal mass. Again, DMAS has detected the presence of the focal mass with the highest SMR, and therefore has been ranked second.

### 4.4. Patient 4

**Figure 8:**
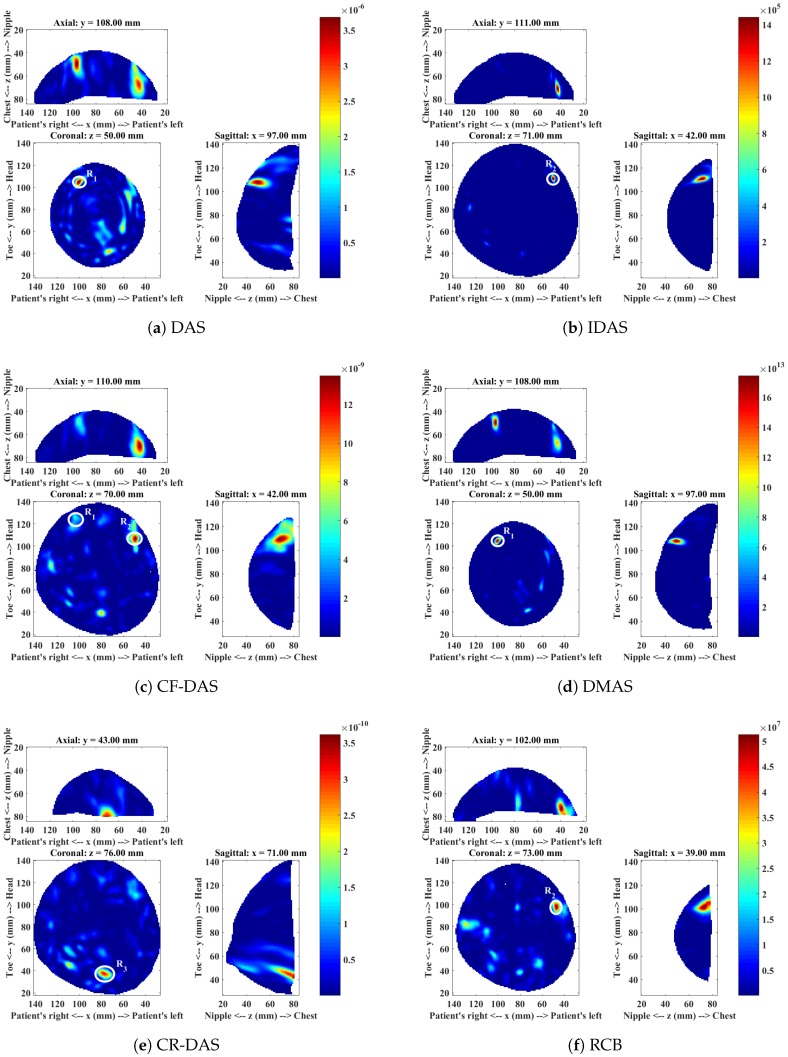
Microwave images of Patient 4 for a range of different algorithms.

The mammogram and the ultrasound report showed a lesion of 11 mm × 7 mm in size at the 10 o’clock position in the left breast. The lesion was later found to be benign with fat necrosis. Figure 8 shows the microwave images produced with all imaging algorithms, and the analysis of the microwave images is presented in Table 5.

The DAS images shows a dominant response $R_{1}$ at a location that could possibly correspond to the location of the lesion. Several other responses with similar high intensities can also be observed in the DAS image. The IDAS image shows the dominant response $R_{2}$ at a location that does not correspond to the lesion location. The response $R_{2}$ is also dominant in the CF-DAS image. The $R_{1}$ lesion can also be observed in the CF-DAS image, but it is relatively weaker in comparison to $R_{2}$. The DMAS image shows the dominant response near to the 10 o’clock position, as was previously observed in the DAS image. The dominant response $R_{3}$ in the CR-DAS image is located closer to the chest wall and does not correspond to any clinically detected lesion. Similar to IDAS and CF-DAS, the RCB image shows dominant response $R_{2}$ that does not correspond to the clinically detected lesion. The DAS and the DMAS images are similar in terms of location of the dominant response. However, the DMAS image has much less clutter than the DAS image. DMAS also provides an improved SMR for the dominant response. In comparison, the IDAS, CF-DAS and RCB images also have much less clutter and a higher SMR of the dominant responses when compared to DAS. However, the dominant response location is inconsistent with the location of the lesion for these three algorithms. The presence of the dominant response closer to the chest wall in the CR-DAS image suggests that CR-DAS has enhanced the clutter as this undesired response does not appear in the images produced with any other algorithm.

Table 5 summarises the detection and image quality metrics computed for each algorithm. DAS and DMAS are the only algorithms that have shown the presence of the benign lesion, with DMAS providing the highest SMR value. The other algorithms have not detected the lesion and therefore have not been considered in the rankings.

### 4.5. Patient 5

**Figure 9:**
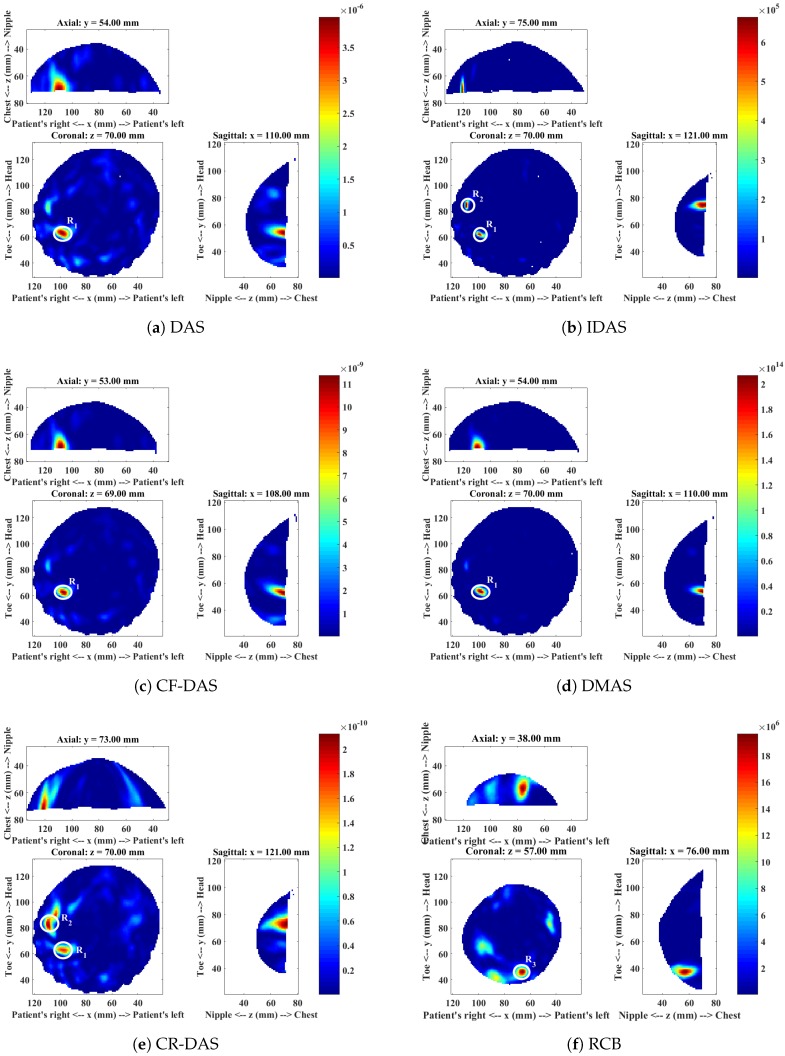
Microwave images of Patient 5 for a range of different algorithms.

Patient 5 had a heterogeneously dense breast, as indicated by the mammography report. The report also indicated a small concentration of fibroglandular tissue on the inner side of the breast and more glandular tissue on the outer side. However, no breast disease was found. The microwave image produced with the DAS (Figure 9a) algorithm shows a dominant response $R_{1}$ near to the 8 o’clock position in the coronal slice. Other weaker responses can also be observed in the DAS image. The dominant response in the image can be attributed to the concentration of fibroglandular tissues, as mentioned in the mammography report. The IDAS image shows two high intensity responses. The response $R_{1}$ is the same response that was dominant in the DAS image. However, a second response $R_{2}$ has appeared near to 9 o’clock, and this response $R_{2}$ is the dominant response in the IDAS image. The CF-DAS and DMAS images show only one response, $R_{1}$. With both CF-DAS and DMAS, $R_{1}$ is not only the dominant response but comparatively the intensity of the second response $R_{2}$ is much weaker. Both responses, $R_{1}$ and $R_{2}$, are present in the CR-DAS image, with $R_{2}$ being the dominant response. The dominant response $R_{3}$ in RCB image does not correspond to any clinically detected lesion.

Both CF-DAS and DMAS significantly improved the SMR of the dominant response $R_{1}$. The higher values of SMR indicate that both algorithms have suppressed clutter in the images. The analysis of the images also indicates that both of the algorithms have not only suppressed the average clutter, but the response $R_{2}$ (that does not correspond to any clinically known region of interest) has also been significantly reduced. The IDAS algorithm improves the SMR of the response $R_{1}$ but it has also improved the SMR of the response $R_{2}$, which wasmuch lower in the DAS image. CR-DAS has produced a similar image to IDAS by improving the SMR of $R_{2}$ and reducing the SMR of $R_{1}$. Finally, RCB exhibits high intensity regions that are not consistent with images produced with other algorithms. Table 6 summarises the detection and image quality metrics computed for each algorithm. There was no tumour present in this patient and the only CI-ROI in this patient was a concentration of fibroglandular tissues. Most algorithms have indicated the presence of a fibroglandular concentration, with the exception of RCB. DMAS provides the highest SMR and has been ranked first in Table 6.

## 5. Discussion

Analysis of the microwave images produced with a variety of imaging algorithms (shown in Figure 3, Figure 4, Figure 5, Figure 6, Figure 7, Figure 8 and Figure 9) indicates that most algorithms demonstrate better background clutter suppression compared to DAS. The IDAS provides the highest SMR value in most cases compared to all other algorithms. However, the responses improved by IDAS often did not correspond to the actual lesion locations (e.g., in case of Patient 1 and Patient 2). Similarly, CF-DAS also provided better clutter suppression, as indicated by an improved average SMR, but the location of dominant responses were found to be inconsistent with the clinical reports. Both of these algorithms improve the dominant response based on the estimated coherence of the responses. However, the coherence metrics for IDAS and CF-DAS may reward responses from non-lesion regions within the breast.

DMAS provided the second highest average SMR value. This indicates a comparable clutter suppression to IDAS, and significantly better than DAS. The location of the dominant responses in DMAS images were consistent with the DAS images, as well as the actual location of lesions reported in clinical reports. In comparison to DAS, DMAS improved the SMR by 44%. The improved performance of the DMAS algorithm can be attributed to its ability to virtually increase the number of measurements, while improving the coherence of responses, using pairing multiplication. Both CR-DAS and RCB performed poorly across all patients in terms of clutter suppression and particularly in terms of the locations of the dominant responses. RCB is known to suffer performance degradations in the presence of coherence interferences [35]. Therefore, the poor performance of RCB can be attributed to the presence of multiple coherent responses and the heterogeneity of the breast. Both CR-DAS and RCB often enhanced the residual clutter from the preprocessing of the signals resulting in incorrect localisation.

It should also be noted that both DAS and DMAS detected a dominant response in the case of Patient 5, even though there was no disease present. This would have resulted in a false-positive detection in the absence of a mammogram. A clear distinction between true-positive and false-positive is a challenge for CMI, which requires further investigations. A number of methods can be employed to define a criterion to reject false-positives. These include comparison of responses from left and right breasts as proposed in [24] and classification of responses into normal and malignant based on the magnitude and shape of target responses [36]. However, large scale clinical studies are required to gather enough data for the development of classification methods to reject false-positive detection.

## 6. Conclusions

In this study, various data independent as well as data adaptive algorithms for confocal microwave breast imaging were applied to patient data. The algorithms were applied in monostatic configuration, however, the relative performance of the algorithms in multistatic configuration is expected to be same. The patient data were obtained from the first patient trials conducted at the University of Calgary. The patients were scanned with the TSAR prototype for microwave imaging, as well as with X-ray mammography and MRI to aid in the interpretation of microwave images.

Results from this study indicate that the conventional DAS is able to detect most malignancies. However, a significant amount of clutter can be observed in the images produced with DAS. The coherence based imaging algorithms (IDAS and CF-DAS) improve the image quality by suppressing the clutter; however, they often fail to correctly localise the malignancy, particularly in the case of multiple lesions and heterogeneous breasts. CR-DAS does not provide significant improvements when compared to DAS. The DA algorithm RCB suffers severe performance degradations in patients, due to the presence of coherent interferences from multiple lesions and the heterogeneity of the breast. DMAS is the only algorithm that consistently demonstrated better clutter suppression and accurate localisation capability compared to all other algorithms.

## Figures and Tables

**Table 1:** Summary of patient information (from [24]).

Patient	Age	Breast Imaged	# of Rows	Measurements per Row	Breast Density	Disease
Patient 1	53	R	6	30	Heterogeneous	Malignancy
Patient 2	64	L	8	20	Extremely dense	Benign
Patient 3	35	L	9	20	Scattered/heterogeneous	Malignancy
Patient 4	44	L	5	30	Heterogeneous	No disease
Patient 5	32	L	6	30	Heterogeneous	No disease

**Table 2:** Summary of the analysis of Patient 1 microwave images.

Imaging Algorithms	ROI		CI-ROI Present in MI?	SMR (dB)	FWHM (mm)	Algorithms Rank
DAS	CI	Malignant lesion ($R_{1}$)	Yes	23.04	32.57	4
	CI	Benign lesion ($R_{2}$)	Yes	21.32	31.19	
	CI	Fibroglandular concentration ($R_{3}$)	Yes	21.39	36.07	
IDAS	CI	Malignant lesion ($R_{1}$)	No	-	-	-
	CI	Benign lesion ($R_{2}$)	Yes	49.92	36.07	
	CI	Fibroglandular concentration ($R_{3}$)	No	-	-	
CFDAS	CI	Malignant lesion ($R_{1}$)	Yes	25.80	27.14	2
	CI	Benign lesion ($R_{2}$)	Yes	29.55	17.23	
	CI	Fibroglandular concentration ($R_{3}$)	Yes	27.16	23.62	
DMAS	CI	Malignant lesion ($R_{1}$)	Yes	39.60	20.04	1
	CI	Benign lesion ($R_{2}$)	Yes	36.27	23.22	
	CI	Fibroglandular concentration ($R_{3}$)	Yes	37.82	17.55	
CRDAS	CI	Malignant lesion ($R_{1}$)	Yes	23.88	31.82	3
	CI	Benign lesion ($R_{2}$)	Yes	27.34	29.20	
	CI	Fibroglandular concentration ($R_{3}$)	No	-	-	
RCB	CI	Malignant lesion ($R_{1}$)	No	-	-	-
	CI	Benign lesion ($R_{2}$)	No	-	-	
	CI	Fibroglandular concentration ($R_{3}$)	No	-	-	
	MI	HI region near to chest wall ($R_{4}$)	-	31.79	15.60	

**Table 3:** Summary of the analysis of Patient 2 microwave images.

Imaging Algorithm	ROI		CI-ROI Present in MI?	SMR (dB)	FWHM (mm)	Algorithm Rank
DAS	CI	Benign lesion ($R_{1}$)	Yes	26.41	13.60	3
IDAS	CI	Benign lesion ($R_{1}$)	No	-	-	-
	MI	HI region close to the chest wall ($R_{2}$)	-	59.56	5.00	
CFDAS	CI	Benign lesion ($R_{1}$)	No	-	-	-
	MI	HI region at the lower outer quadrant of the breast ($R_{3}$)	-	35.48	10.72	
DMAS	CI	Benign lesion ($R_{1}$)	Yes	45.66	8.78	1
CRDAS	CI	Benign lesion ($R_{1}$)	Yes	30.06	13.34	2
RCB	CI	Benign lesion ($R_{1}$)	No	-	-	-
	MI	HI region below the nipple ($R_{4}$)	-	38.03	6.96	

**Table 4:** Summary of the analysis of Patient 3 microwave images.

Imaging Algorithm	ROI		CI-ROI Present in MI?	SMR (dB)	FWHM (mm)	Algorithm Rank
DAS	CI	Malignant tumour	No	-	-	4
	CI	Focal mass ($R_{1}$)	Yes	28.38	17.11	
	MI	HI region near to the nipple ($R_{2}$). Probably part of the focal mass.		24.62	20.15	
IDAS	CI	Malignant tumour ($R_{3}$)	Yes	58.74	5.74	1
	CI	Focal mass ($R_{1}$)	No	-	-	
CFDAS	CI	Malignant tumour	No	-	-	3
	CI	Focal mass ($R_{1}$)	Yes	38.89	11.35	
	MI	HI region near to the nipple ($R_{4}$).	-	43.89	11.05	
DMAS	CI	Malignant tumour	No	-	-	2
	CI	Focal mass ($R_{1}$)	Yes	52.11	11.58	
CRDAS	CI	Malignant tumour	No	-	-	-
	CI	Focal mass ($R_{1}$)	No	-	-	
	MI	HI region at 4’o clock ($R_{5}$)	-	30.75	20.92	
RCB	CI	Malignant tumour	No	-	-	-
	CI	Focal mass ($R_{1}$)	No	-	-	
	MI	HI region near to the nipple ($R_{4}$)		56.78	9.06	

**Table 5:** Summary of the analysis of Patient 4 microwave images.

Imaging Algorithm	ROI		CI-ROI Present in MI?	SMR (dB)	FWHM (mm)	Algorithm Rank
DAS	CI	Benign lesion ($R_{1}$)	Yes	22.04	25.40	2
IDAS	CI	Benign lesion ($R_{1}$)	No	-	-	-
	MI	HI region near to 2’o clock (R2)	-	54.55	11.18	
CFDAS	CI	Benign lesion ($R_{1}$)	No	-	-	-
	MI	HI region near to 2’o clock (R2)	-	27.26	28.17	
DMAS	CI	Benign lesion ($R_{1}$)	Yes	39.81	11.04	1
CRDAS	CI	Benign lesion ($R_{1}$)	No	-	-	-
	MI	HI region closer to the chest wall (*R*_3_)	-	29.60	24.51	
RCB	CI	Benign lesion ($R_{1}$)	No	-	-	-
	MI	HI region near to 2’o clock ($R_{2}$)	-	33.15	15.30	

**Table 6:** Summary of the analysis of Patient 5  microwave images.

Imaging Algorithm	ROI		CI-ROI Present in MI?	SMR (dB)	FWHM (mm)	Algorithm Rank
DAS	CI	Fibroglandular concentration ($R_{1}$)	Yes	28.20	17.11	4
IDAS	CI	Fibroglandular concentration ($R_{1}$)	Yes	48.65	8.18	2
	MI	HI region near to 9 o’ clock ($R_{2}$)	-	49.15	9.49	
CFDAS	CI	Fibroglandular concentration ($R_{1}$)	Yes	34.88	14.14	3
DMAS	CI	Fibroglandular concentration ($R_{1}$)	Yes	49.37	10.49	1
CRDAS	CI	Fibroglandualar concentration ($R_{1}$)	Yes	23.61	20.80	5
	MI	HI region near to 9 o’clock ($R_{2}$)	-	25.03	27.77	
RCB	CI	Fibroglandular concentration (*R*_1_)	No	-	-	-
	MI	HI region near to 6 o’clock ($R_{3}$)	-	30.72	12.04	
